# Human skin triglycerides prevent bed bug (*Cimex lectularius* L.) arrestment

**DOI:** 10.1038/s41598-021-01981-1

**Published:** 2021-12-08

**Authors:** Sudip Gaire, Zachary C. DeVries, Russell Mick, Richard G. Santangelo, Grazia Bottillo, Emanuela Camera, Coby Schal

**Affiliations:** 1 Department of Entomology, University of Kentucky, 1100 S. Limestone, S-225 Ag North, Lexington, KY 40546-0091 USA; 2 Department of Entomology and Plant Pathology, North Carolina State University, Raleigh, NC USA; 3 Laboratory of Cutaneous Physiopathology, San Gallicano Dermatological Institute-IRCCS, Rome, Italy

**Keywords:** Behavioural ecology, Chemical ecology, Lipids, Entomology

## Abstract

Bed bugs (*Cimex lectularius*) have proliferated globally and have become one of the most challenging pests to control indoors. They are nocturnal and use multiple sensory cues to detect and orient towards their human hosts. After feeding, usually on a sleeping human, they return to a shelter on or around the sleeping surface, but not directly on the host. We hypothesized that although human skin odors attract hungry bed bugs, human skin compounds may also prevent arrestment on hosts. We used arrestment assays to test human skin swabs, extracts from human skin swabs, and pure compounds identified from human skin swabs. When given a choice, bed bugs preferred to arrest on substrates not previously conditioned by humans. These responses were consistent among laboratory-reared and apartment-collected bed bugs. The compounds responsible for this behavior were found to be extractable in hexane, and bed bugs responded to such extracts in a dose-dependent manner. Bioassay-guided fractionation paired with thin-layer chromatography, GC–MS, and LC–MS analyses suggested that triglycerides (TAGs), common compounds found on human skin, were preventing arrestment on shelters. Bed bugs universally avoided sheltering in TAG-treated shelters, which was independent of the number of carbons or the number of double bonds in the TAG. These results provide strong evidence that the complex of human skin compounds serve as multifunctional semiochemicals for bed bugs, with some odorants attracting host-seeking stages, and others (TAGs and possibly other compounds) preventing bed bug arrestment. Host chemistry, environmental conditions and the physiological state of bed bugs likely influence the dual nature behavioral responses of bed bugs to human skin compounds.

## Introduction

Bed bugs (*Cimex lectularius* L.) are obligate hematophagous pests that live in close proximity to humans^1^. Since their global resurgence in the early 2000s, bed bugs have become one of the most challenging insects to control indoors. Bed bugs have also been shown to adversely affect human health, causing allergic reactions to their bites^2^, psychological distress associated with both past and present infestations^3,4^, and contamination of the indoor environment with histamine and various microbes^5,6^.

Unlike some hematophagous arthropods that have formed close associations with their hosts (e.g., lice, fleas), bed bugs do not rest or reside on their host, but instead shelter in close proximity to the host^1^. Therefore, they are typically found in areas where their host is located at night (e.g., bed, sofa) and their host-seeking activities generally range over relatively short distances (< 1 m)^1^. Bed bugs use the same set of cues commonly used by other hematophagous arthropods, namely CO_2_, heat, and chemosensory cues^7^. The precise role of CO_2_ is equivocal, as some studies have found it to be an attractant that provides directional information^8–11^, whereas a recent study suggests that in large arenas (the size of a room) CO_2_ may only serve to activate bed bugs from an arrested state^12^. Heat is responsible for activation, orientation, and feeding^13,14^, with the terminal tip of the antenna guiding these behavioral responses^15^. Human odors have been shown to be attractive to bed bugs^12^, and independently of other cues can guide bed bugs in olfactometers^16^. Thus, several sensory modalities and multiple cues are integrated in host-seeking bed bugs, with behavioral responses being modulated by both environmental factors (e.g., ambient CO_2_)^12^ and physiological states (e.g., mating and starvation)^17^.

After feeding (or when the photophase begins if they were unsuccessful in obtaining a blood meal during the scotophase), bed bugs switch from host- to shelter-seeking behaviors^1^. Shelter- or aggregation-seeking is guided by an aggregation pheromone^18,19^, with some evidence that bed bugs may follow trails to and from their hosts^20^. Physiological factors such as age and sex can also influence the aggregation behavior of bed bugs in shelters^21^, where the propensity of adult females to aggregate is comparatively lower than of adult males and nymphs^22,23^. When mated and unmated females were compared for their aggregation activity on shelters treated with putative volatile aggregation pheromone components, only unmated females were attracted^24^. The tendency to aggregate also decreases with starvation^25^.

Although many factors influence aggregation, bed bugs have not been reported to arrest on their host, but instead they shelter in close proximity to the host^1^. This suggests a dual nature of host cues, which simultaneously attract bed bugs and prevent their arrestment on the host, likely dependent upon environmental and physiological factors of the bed bug (e.g., feeding status, mating status, etc.) and the host (e.g., skin compounds, host body temperature, CO_2_ levels, host movement, etc.). Since attractive host odors emanate from human skin^16^, and skin contains a complex chemical profile of volatile and non-volatile compounds^26,27^, we hypothesized that some of these compounds may be responsible for preventing arrestment on human hosts. Using arrestment bioassays, we evaluated bed bug behavior in shelters treated with human skin extracts, followed by bioassay-guided fractionation paired with thin-layer chromatography (TLC), gas-chromatography-mass spectrometry (GC–MS), and liquid-chromatography-tandem MS (LC–MS) analyses to identify human skin compounds that prevent bed bug arrestment.

## Materials and methods

### Bed bugs

Four bed bug populations (one laboratory strain and three collected from infested homes) were used in this study (Table 1). All populations were reared in the laboratory as described by DeVries et al.^28^. Briefly, bed bugs were maintained in 168 cm^3^ plastic containers on paper substrate at 25 °C, 50% relative humidity, and a photoperiod of 12 h:12 h (Light:Dark). Bed bugs were fed defibrinated rabbit blood (Hemostat Laboratories, Dixon, CA, USA) weekly using an artificial feeding system. This system maintained blood at 35 °C by circulating water through custom-made water-jacketed glass feeders. An artificial membrane (plant budding tape, A.M. Leonared, Piqua, OH, USA) was stretched over the bottom of each glass feeder, containing the blood while simultaneously allowing bed bugs to feed through it. In all experiments, adult males starved for 7–10 days were used. All populations were used for documenting responses to human skin swabs. The WS population was used for bioassays with various human volunteers and hexane extracted swabs, and the JC population was used for testing various lipids.

**Table 1:** Bed bug populations used in this study.

Name	Area of collection	Date of collection
Harold Harlan (HH)^a^	Ft. Dix, NJ, USA	1973
Winston Salem (WS)^b^	Winston Salem, NC, USA	2008
Jersey City (JC)^b^	Jersey City, NJ, USA	2008
Courtyard (CC)^b^	Raleigh, NC, USA	2009

^a^Standard laboratory population.

^b^Apartment-collected population.

### Skin swab collection

The North Carolina State University Institutional Review Board approved this study (IRB #14173). Informed consent was obtained from all human participants, and all the methods were performed according to the relevant guidelines and regulations. Six human volunteers (3 males, 3 females) ranging from 25 to 50 years old representing several ethnicities (white/Caucasian, Hispanic, Asian) provided samples for this project. Skin swabs were collected following the exact methods outlined by DeVries et al.^16^. In our 2019 study, these swabs were reported to attract bed bugs independent of other cues in Y-tube olfactometer assays. Briefly, participants were asked to follow a standard operating procedure, which was reviewed with them prior to sample collection. Before collecting skin swabs, participants were asked to not to eat ‘spicy’ food for at least 24 h, take a morning shower, avoid the use of deodorant and cosmetics after showering, and avoid strenuous physical activity. Skin swabs were collected 4–8 h after showering. Hands were washed with water only before lifting filter paper. Swabs were collected using 4.5 cm diameter filter paper discs (#1; Whatman plc, Madistone, United Kingdom). Both sides of a single filter paper disc were rubbed over the left arm from hand to armpit for 12 s, left leg from lower thigh to ankle for 12 s, and left armpit for 6 s. This procedure was repeated on the right side using a new filter paper disc, so that two samples were collected during each swabbing session. The skin swab samples were then stored in glass vials at − 20 °C, and used within one month of collection. The swabs from all human volunteers were used to compare participants and establish that bed bugs responded similarly to all, and participant A’s skin swabs were used for all subsequent bioassays.

### Two-choice arrestment bioassays

**Figure 1:**
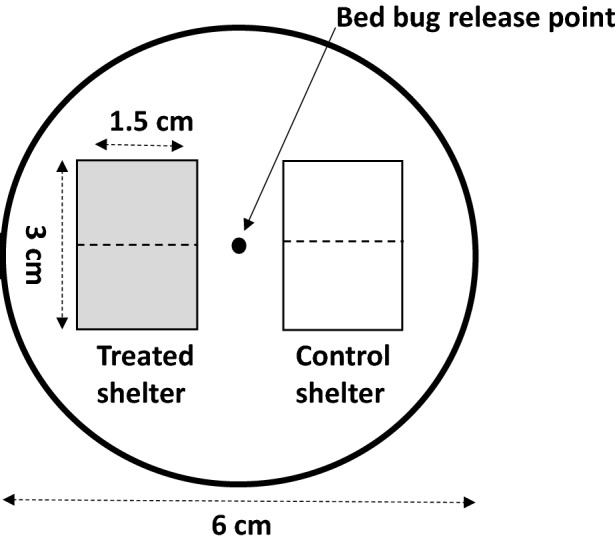
Two-choice behavioral assay (top-view) consisting of two equal size paper shelter tents. A clean filter paper (control) was always paired with a treated filter paper that either represented a human skin swab, hexane extract of swabbed paper, SPE fraction of human skin swab extract, or authentic TAGs. A single male bed bug was introduced into the center of each arena and allowed to select a tent to arrest under.

Two-choice bioassays were conducted in plastic Petri dishes of 6 cm diameter (Corning Life Sciences, Durham, NC, USA) (Fig. 1). The bottom surface of each Petri dish was roughened so that bed bugs could freely move about the arena. Two tents (3 × 1.5 cm) were created using filter paper (Whatman #1). One tent served as the control tent, and the other served as the treatment tent. Control tents were either untreated (nothing added) or treated with hexane only. Treatment tents were either made directly from human odor swabs, treated with human odor extract (in hexane), or treated with a specific compound (in hexane). Tents were allowed 60 min to acclimate to room conditions and allow for the solvent to evaporate prior to initiating bioassays. The positions of tents (treatment and control) were alternated to account for any side-bias.

Adult male bed bugs were housed in individual vials for 24 h prior to each experiment. A single adult male bed bug was released in the middle of the arena 5 h into the scotophase, by transferring it on its harborage. The harborage material was removed immediately after the bed bug moved off of it (the harborage). Bed bugs were allowed the remaining 7 h of the scotophase to freely move around the arena, with their final position reported 3 h into the photophase. Bed bugs that were in contact with the filter paper with any part of their body were recorded as making a choice (i.e. arrestment state); others not in contact with either filter paper tent were recorded as non-responders, reported in the figures, but not used in data analysis. It should be noted that momentary pauses in movement (feeding or other behaviors) are not referred to as arrestment in this study. In total, 15–39 replicates were performed for each experiment (reported for each bioassay).

### Bioassays with human skin swabs

Bioassays with human skin swabs were performed to understand if bed bug arrestment behavior (1) differed among different bed bug populations, and (2) influenced by different host odors. Skin swabs were removed from the freezer, equilibrated to room temperature, divided into three equal parts and trimmed to a rectangular shape corresponding to the size of a shelter tent (Fig. 1). Skin swabs from participant A were used to evaluate the responses of four bed bug populations (Table 1). Skin swabs from all participants A–F were used to evaluate the robustness of our findings across multiple human hosts.

### Skin swab extraction and fractionation

Skin swabs collected from volunteer A were pooled and extracted in hexane. Extraction procedures were carried out sequentially by placing a single skin swab into a 20 ml glass vial containing 5 ml of hexane, vortexing for 30 s, then moving the filter paper to a new 20 ml vial containing 5 ml of hexane and repeating the process. Three sequential extractions were performed for each skin swab, and a minimum of 10 skin swabs (collected over several days) were used for each extraction. After all skin swabs were extracted, all sequential hexane extracts were combined and concentrated to a final concentration of one skin swab equivalent per 300 µl, or one bioassay equivalent (BE) per 100 µl (since each swab was used for 3 bioassays; see “Bioassays with human skin swabs” for more information on the size used for each bioassay). Control swabs were also extracted. These swabs were treated identically to the skin swabs, except they did not contact human skin.

To determine what compound classes were responsible for the observed behavior, hexane extracts were fractionated using solid phase extraction (SPE). Extracted samples were concentrated to 1 BE/10 µl hexane, then loaded onto a 1 g silica SPE column (6 ml total volume; J.T. Baker, Phillipsburg, NJ, USA). The column was eluted with the following solvents (4 ml of each, each repeated twice sequentially): hexane, 2% ether (in hexane), 5% ether (in hexane), 10% ether (in hexane), 20% ether (in hexane), 50% ether (in hexane), 100% ether, ethyl acetate, and methanol (all solvents acquired from Sigma Aldrich, St. Louis, MO, USA). Each solvent fraction was then concentrated to a final concentration of 1 BE/100 µl and stored at − 20 °C.

### Bioassays with extracted and fractionated human skin swabs

For all extraction and fractionation bioassays, filter paper tents were cut to a size of 3 cm × 1.5 cm (Fig. 1) and treated with 100 µl (1 BE) of extracted or fractionated human skin swabs (50 µl on each side). A dose–response bioassay was run first to determine if the compounds responsible for bed bug arrestment responses could be extracted and at what concentration (BE) they were behaviorally active. Dilutions were made in hexane, with control tents receiving extracts of control filter paper. At least 20 replicates were conducted for each concentration. After validating an appropriate BE that could be used in future experiments, SPE fractions were diluted in hexane to 0.1 BE and applied to filter paper tents as previously described (50 µl per side). A minimum of 15 replicates were conducted for each fraction to identify behaviorally active fractions.

### Compound identification

To better understand what classes of compounds were present in behaviorally active fractions, we conducted thin layer chromatography (TLC) with known standards. A flexible, silica (250 µm) TLC plate (Whatman) was placed into a glass chamber containing a solvent layer of 1.5 cm. The plate was cleaned twice with acetone, then standards (triglyceride [TAG], wax ester, squalene) and samples (fractions) were each loaded into separate lanes. The plate was developed twice in 10% ether (in hexane), then visualized non-destructively with iodine.

In addition, behaviorally active fractions were further evaluated for their composition with GC–MS and LC–MS. GC–MS was employed to analyze free fatty acids, squalene, and cholesterol^29^, whereas LC–MS was employed to characterize the intact skin lipids as previously described^30^. Samples were analyzed with a GC 7890A coupled to the MS 5975 VL analyzer (Agilent Technologies, CA, USA) following derivatization. Briefly, 50 µL of the extract dissolved in isopropanol were dried under nitrogen and derivatized with 100 µL BSTFA containing 1% trimethylchlorosilane (TCMS) in pyridine to generate the trimethylsilyl (TMS) derivatives at 60 °C for 60 min. GC separation was performed with a 30 m × 0.250 mm (i.d.) × 0.25 µm film thickness DB-5MS fused silica column (Agilent). Helium was used as the carrier gas. Samples were acquired in scan mode by means of electron impact (EI) MS.

Liquid-chromatography coupled to the MS analyzer by means of an electrospray interface (ESI) was used to determine abundance and ESI tandem MS of non-volatile lipids as previously described^29,30^. LC separation was performed with a reverse phase Zorbax SB-C8 column (2.1 × 100 mm, 1.8 μm particle size, Agilent). Data were acquired in the positive ion mode at unit mass resolving power by scanning ions between m/z 100 and 1000 with G6410A series triple quadrupole (QqQ) (Agilent). LC runs and MS spectra were processed with the Mass Hunter software (B.09.00 version).

### Bioassays with triglycerides

After determining that TAGs were prominent compounds in bioactive skin swab fractions, commercially available TAGs were evaluated for behavioral activity. Filter paper tents were treated with 100 µl of hexane (50 µl to each side) containing TAG standards. First, tripalmitin (16:0/16:0/16:0) (Sigma-Aldrich) was evaluated in a dose–response fashion (60 µg to 0.6 µg) to determine what level of TAG was appropriate for bioassays. The upper level of testing was set at 60 µg as a conservative estimate of the amount of TAGs bed bugs may be exposed to, based on calculations of our arena size and previous reports of TAGs on human skin and sebum. Specifically, previous reports documented that 1.5 mg of sebum could be passively collected using Sebutape from an area of 4.7 cm^2^^30,31^. Because TAGs typically constitute 60% of human sebum^32^, it is reasonable to assume that passive collection of sebum can result in > 190 µg/cm^2^ of TAGs in a short amount of time (30 min). Our sampling methods involved swabbing rather than passive collection, but our use of 60 µg over a 9 cm^2^ (two sides of 4.5 cm^2^) shelter tent (6.67 µg/cm^2^) is a low-estimate of the amount of TAGs collected (although this was not directly measured in the current study). Other TAGs that we tested at a concentration of 60 µg per 9 cm^2^ included the saturated TAGs trimyristin (14:0/14:0/14:0) and tristearin (18:0/18:0/18:0) and the unsaturated TAGs triolein (18:1/18:1/18:1), trilinolein (18:2/18:2/18:2), and trilinolenin (18:3/18:3/18:3) (all from Sigma-Aldrich). A minimum of 30 replicates were conducted with each TAG.

### Statistical analysis

A Chi-square goodness of fit test was used to compare the responses of bed bugs to control versus treated tents in all two-choice bioassays, with the null hypothesis that if bed bugs do not respond differentially to treated tents they should display a 1:1 preference ratio for both sides of the assay. All tests were conducted in SPSS Version 26 (IBM Corp., Armonk, NY).

## Results

### Bioassays with human skin swabs

**Figure 2:**
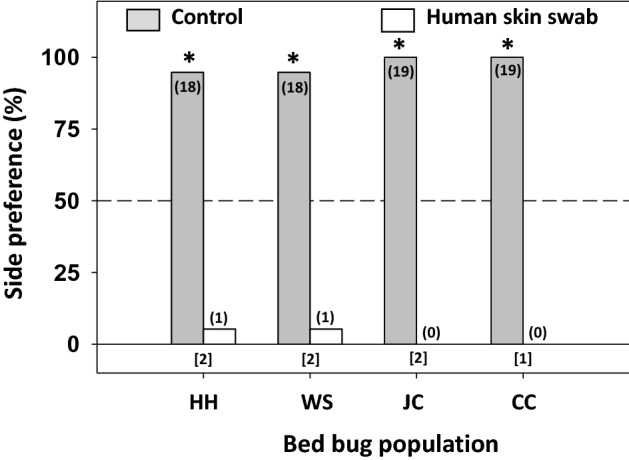
Responses of four bed bug populations to human skin swabs. An asterisk (*) indicates a significant preference for one side of the two-choice assay (Chi-square test, *P* < 0.05). The number of bed bugs that responded is indicated in parentheses for each choice, and the number of bed bugs that failed to respond (i.e., rested on neither tent) is indicated in brackets below each set of choices for each assay. HH = Harold Harlan, WS = Winston Salem, JC = Jersey City, CC = Courtyard.

**Figure 3:**
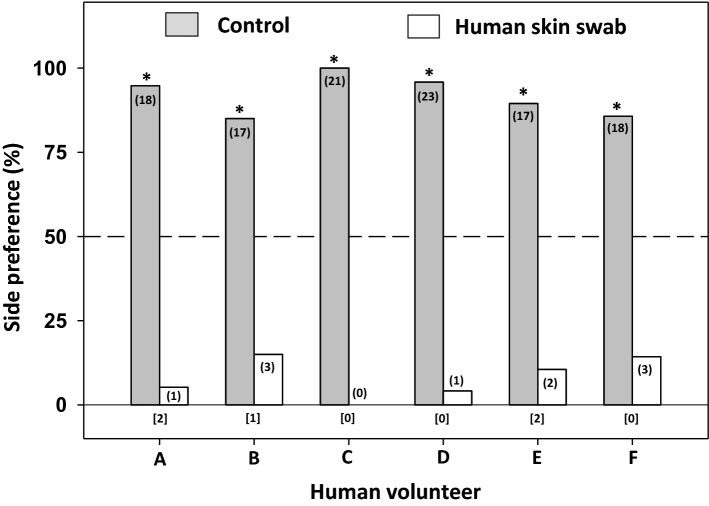
Responses of bed bugs to human skin swabs from different human volunteers. An asterisk (*) indicates a significant preference for one side of the two-choice assay (Chi-square test, *P* < 0.05). The number of bed bugs that responded is indicated in parentheses for each choice, and the number of bed bugs that failed to respond (i.e., rested on neither of the two shelters) is indicated in brackets below each set of choices for each assay.

All four bed bug populations (one laboratory-reared and three more recently apartment-collected) showed significant preferences for the control (untreated) filter paper (population HH: χ^2^_1,19_ = 15.21; WS: χ^2^_1,19_ = 15.21; JC: χ^2^_1,19_ = 19.00; CC: χ^2^_1,19_ = 19.00; all *P* < 0.001; Fig. 2), with > 94% of adult males from all populations selecting the control tents. These results indicate that compounds from human skin prevent arrestment, and that these responses are consistent among different bed bug populations. In addition, bed bugs showed consistent behavioral avoidance of skin swabs obtained from six different human volunteers, with significant preferences for the control tent regardless of the source of the skin swab (participant A: χ^2^_1,19_ = 15.21, *P* < 0.001; B: χ^2^_1,20_ = 9.80, *P* = 0.002; C: χ^2^_1,21_ = 21, *P* < 0.001; D: χ^2^_1,24_ = 20.16, *P* < 0.001; E: χ^2^_1,19_ = 11.84, *P* = 0.001; F: χ^2^_1,21_ = 10.71, *P* = 0.001; Fig. 3).

### Responses of bed bugs to hexane extracted skin swabs

**Figure 4:**
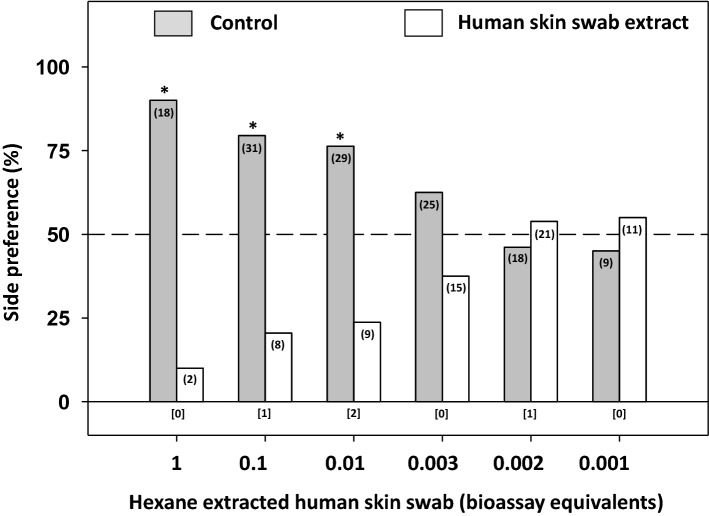
Responses of bed bugs to hexane extracted filter paper skin swabs at different concentrations. One bioassay equivalent is equal to the amount of material present in a standard assay (before extraction). An asterisk (*) indicates a significant preference for one side of the two-choice assay (Chi-square test, *P* < 0.05). The number of bed bugs that responded is indicated in parentheses for each choice, and the number of that did not respond (i.e., rested on neither of the two shelters) is indicated in brackets below each set of choices for each assay.

Bed bugs responded to hexane-extracted skin swabs in a dose-dependent manner (Fig. 4). Bed bugs showed significant avoidance of hexane-extracted skin swabs of the following concentrations: 1 BE (1/3 of a single swab) (χ^2^_1,20_ = 12.80, *P* < 0.001), 0.1 BE (χ^2^_1,39_ = 13.56, *P* < 0.001) and 0.01 BE (χ^2^_1,38_ = 10.52, *P* = 0.001) (Fig. 4). At concentrations < 0.01 BE there were no significant preferences for either treated or control filter papers (Fig. 4).

### Responses of bed bugs to fractions of human skin swab extracts

**Figure 5:**
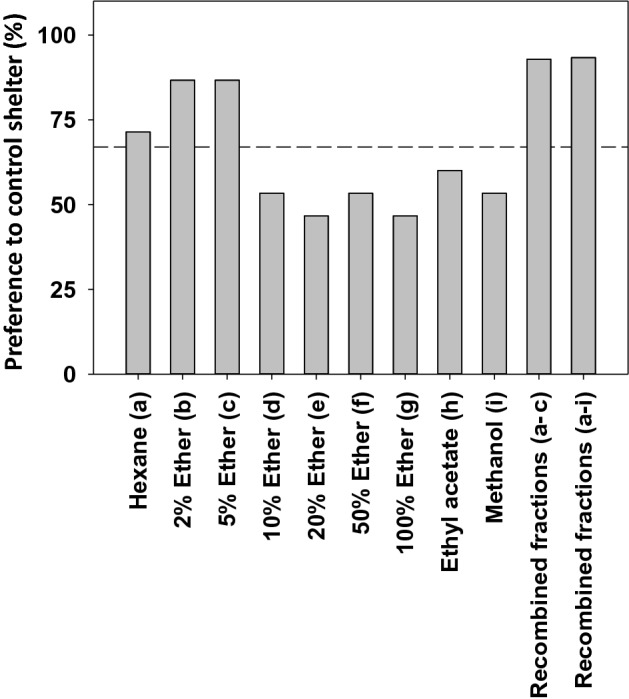
Response of bed bugs in fractionated (SPE) human skin swab extracts. Bed bug preference for the control shelter is reported from the screening assays. Fractions in which > 67% of bed bugs chose the control shelter were evaluated further. Except in hexane and the recombined fractions (a–c; non-responders = 6.66%), all bugs made a choice (100% response rate). In total, 15 insects were used for each fraction tested.

Skin swab extracts were fractionated on normal phase SPE (silica gel). Bioassays revealed that active compounds were present in the following fractions: hexane, 2% ether (in hexane), and 5% ether (in hexane) (Fig. 5). This process was used as a screening tool, and thus a threshold of > 67% preference for the control shelter was used to select fractions for further evaluation.

### Human skin triglycerides

**Figure 6:**
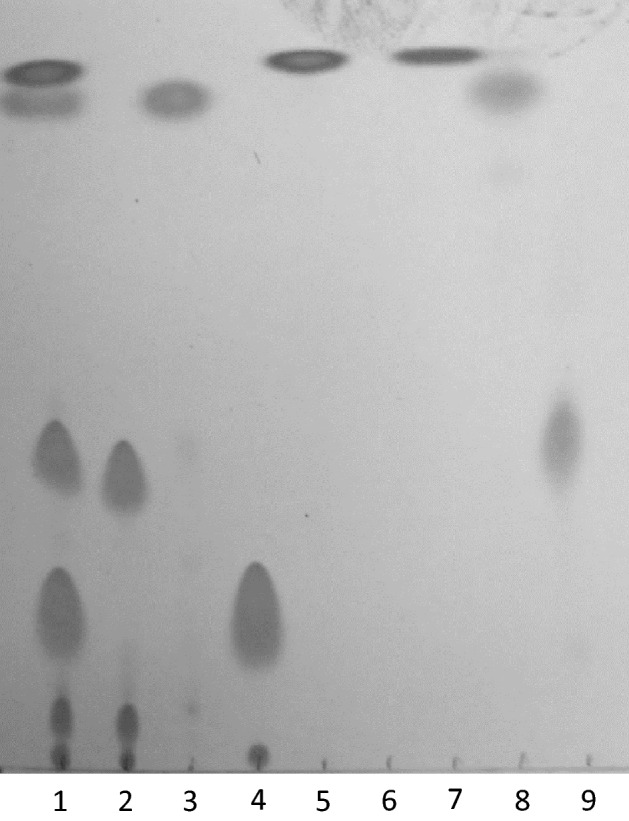
Thin layer chromatogram of human skin swab extract fractionated on a normal phase silica gel plate. Lane assignments are as follows: 1—All standards (trilinolein [20 µg], jojoba oil [20 µg], linoleic acid [20 µg], squalene [10 µg]), 2—trilinolein (20 µg), 3—jojoba oil (20 µg), 4—linoleic acid (20 µg), 5—squalene (10 µg), 6—blank, 7—hexane fraction (0.1 BE), 8—2% ether (in hexane) fraction (0.1 BE), 9—5% ether (in hexane) fraction (0.1 BE).

Thin layer chromatography identified several classes of compounds present in the bioactive fractions. Specifically, we identified TAGs in the 5% ether (in hexane) fraction, wax esters in the 2% ether (in hexane) fraction, and squalene in the hexane fraction (Fig. 6). The combined GC–MS and LC–MS analyses confirmed the TLC results. In general, the hexane fraction contained high amounts of squalene; the 2% ether fraction contained high amounts of squalene epoxide in addition to wax esters and cholesterol esters; and the 5% ether fraction contained high amounts of TAGs and free fatty acids. TAGs were further characterized by subjecting the 5% ether fraction to MS–MS fragmentation. Product ion scanning of TAGs showed that fatty acid chain lengths between 14 and 18 C-atoms were the most represented in our samples (Tables S1 and S2). Although several TAGs were detected in the analyses, we chose to focus on compounds that were commercially available.

### Response of bed bugs to selected triglycerides

**Figure 7:**
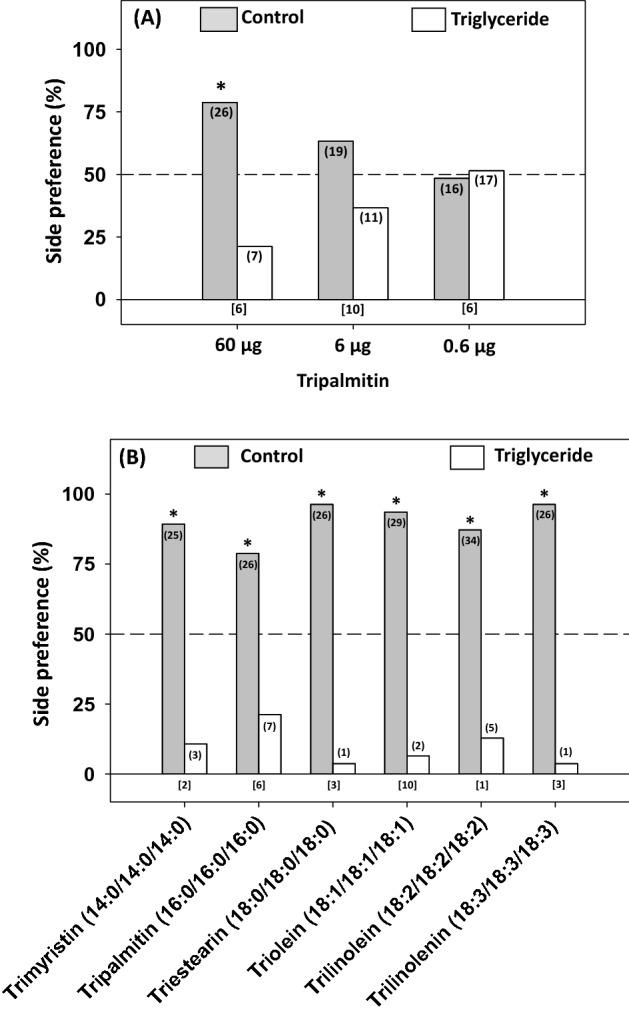
Response of bed bugs to various lipids. (**A**) Bed bugs responded to tripalmitin in a dose-dependent manner. (**B**) Various triglycerides (at 60 µg) of different compositions prevented bed bug arrestment. An asterisk (*) indicates a significant preference for one side of the two-choice assays (Chi-square test, *P* < 0.05). The number of bed bugs that responded is indicated in parentheses for each choice, and the number of bed bugs that did not respond (did not contact either of the two shelters) is indicated in brackets below each set of choices for each assay.

Tripalmitin prevented bed bugs from arresting in a dose-dependent manner (Fig. 7A). Bed bugs showed a significant preference for control tents over tents treated with 60 µg of tripalmitin (χ^2^_1,33_ = 10.93, *P* = 0.001). This preference declined as the dose of tripalmitin decreased to 6 µg and 0.6 µg, neither of which resulted in significant side preference (*P* > 0.5) (Fig. 7A). Therefore, a dose of 60 µg was used in bioassays with other TAGs.

Bed bugs avoided shelters treated with all six TAGs that included both saturated and unsaturated compounds ranging from C42 to C54—trimyristin (χ^2^_1,28_ = 17.28), tripalmitin (χ^2^_1,33_ = 10.93), triestearin (χ^2^_1,27_ = 23.14), triolein (χ^2^_1,31_ = 25.51), trilinolein (χ^2^_1,39_ = 21.56) and trilinolenin (χ^2^_1,27_ = 23.14; all *P* < 0.001) (Fig. 7B)—indicating that neither carbon chain length (between 14 and 18 carbons) nor the number of double bonds (0–3) affected the behavioral responses elicited by these compounds.

## Discussion

This study shows that compounds found on human skin can prevent bed bugs from arresting on conditioned surfaces. This proved true for different populations of bed bugs, and for bed bugs tested with skin swabs collected from different human volunteers. Bed bugs responded to extracts from human skin swabs in a dose-dependent manner, confirming that compounds present on human skin were responsible for preventing arrestment on treated shelters. Further, using bioassay-guided fractionation, we identified several behaviorally active fractions. After subjecting behaviorally active fractions to TLC and detailed verification by GC–MS and LC–MS analyses, we determined that TAGs were present in our samples and likely served as semiochemicals.

Triglycerides are tri-esters derived from glycerol and three fatty acids^33^. They can be found in skin lipids of humans primarily derived from two sources: the sebaceous glands and epidermis^26^. Relative to other skin lipid compounds (e.g., squalene, wax esters, sterol esters, and saturated hydrocarbons), TAGs are found in relatively high amounts^26^, which is consistent among humans of different ages, gender, and ethnicity^27,34^. These analytical findings correlated with our bioassay results where bed bugs also did not show any bias in their behavior towards skin swabs of human volunteers of different ages, gender, and ethnicity.

Several authentic TAGs effectively prevented arrestment behavior in bed bugs. Although we tested only a small number of TAGs, carbon chain length (14–18) and number of double bonds (0–3) in their constituent fatty acids did not affect bed bug behavioral responses. Triglycerides are relatively large compounds which are not volatile at room temperature. Bed bugs detect cues (mostly volatile) using sensilla on the antennae and other sensory appendages. The distal tip of each antenna houses seven different sensilla, with type-Ds shown to be responsible for human odorant reception^35–37^. A more detailed single sensillum electrophysiology study is needed to investigate the roles of various sensilla in detecting TAGs. We suspect that gustatory sensilla may also be involved.

Independent of other host cues, human skin compounds (collected via skin swabs) serve two independent functions in bed bug behavior. Our results provide the first insight as to why hungry bed bugs orient toward human odors^12,16^, but then seek shelter away from the host. Given that our skin swab methods were identical to those employed by DeVries et al.^16^, it is interesting that with a simple change in bioassay procedures (Y-tube olfactometer to shelter-based arrestment assay), bed bug behavior switched from attraction to refusal to arrest. These findings suggest that volatile compounds are responsible for host attraction, while non-volatile (or low-volatility) compounds prevent arrestment. Furthermore, this paradigm provides a beneficial feedback loop for bed bugs, ensuring that they find their host, but do not remain on their host where they likely experience lower survival given they are not adapted for living on their hosts. It should also be noted that other stimuli (e.g., body temperature, CO_2_ levels, host movement, etc.) may also deter arrestment, but these stimuli were not evaluated in the current study.

In the current study, bed bug behavior was not measured in real time (e.g., video tracking), so we cannot quantify attraction to shelters. However, we observed free movement between both shelters (personal observation), suggesting that TAGs do not interfere with attraction to the shelter and likely they are not repellent, but rather they prevent bed bugs from arresting on the shelter. It is noteworthy that attraction and arrestment are also driven by two different sets of compounds in shelter-seeking bed bugs. Volatile aggregation pheromone components, derived from sternal glands and feces, attract bed bugs to shelters, whereas arrestment is driven by histamine in bed bug feces^18^. The interplay between histamine and TAGs would be worth investigating in future studies.

Likewise, it would be interesting to determine how physiological and environmental factors drive the switch from host-seeking to shelter-seeking, and thus a preference for locations without TAGs. In addition, given the similarities in host-seeking behaviors among hematophagous arthropods, it is possible that TAGs or other similar skin compounds may drive anti-arrestment behavior in other blood-sucking arthropods, especially hemipterans that do not arrest on their host.

Even when bed bugs did not take a blood meal, they still refused to arrest on shelters treated with human skin swabs. This is notable, because starvation is a critical factor that affects various aspects of bed bug biology, including host-seeking behavior^17,38^, aggregation behavior^25^, metabolism^39^, and insecticide resistance^40^. In our assays, male bed bugs starved for 7–10 days consistently refused to arrest on human skin swabs, despite being in a host-seeking state^13,15,16^. This indicates the human skin compounds prevent bed bugs from arresting on conditioned surfaces independent of feeding status (up to 10 days, longer periods of starvation were not tested).

## Conclusion

This study demonstrates that substrates conditioned by human skin or treated with compounds from human skin can prevent bed bug arrestment. Of the many compounds found on human skin, TAGs were capable, independently, of preventing arrestment. Future studies should evaluate TAG-induced anti-arrestment behavior in other bed bug life stages and in bed bugs of different physiological states (fed, starved, mated, unmated, etc.) using multiple assays, including continuous live video tracking, to further unravel this unique phenomenon where two disparate cues are encoded in one matrix. Furthermore, other hematophagous species should be evaluated to determine if this behavior is unique to bed bugs. Detailed molecular and electrophysiology studies should also be implemented to better understand the mechanism of TAG detection by bed bugs. The TAG compounds identified in this study may be applicable to future management efforts, given the strong anti-arrestment behavior they elicit.

## Supplementary Information

Supplementary Tables.
